# Complete Genome Sequence of a Rabies Virus Isolated from a Human in Central African Republic

**DOI:** 10.1128/genomeA.00598-14

**Published:** 2014-06-19

**Authors:** Vianney Tricou, Nicolas Berthet, Emmanuel Nakouné, Mirdad Kazanji

**Affiliations:** a Department of Virology, Institut Pasteur de Bangui, Bangui, Central African Republic; b Epidemiology and Physiopathology of Oncogenic Viruses, Institut Pasteur, Paris, France; c UMR3569, Centre National de la Recherche Scientifique, Paris, France; d National Laboratory for Rabies, Institut Pasteur de Bangui, Bangui, Central African Republic

## Abstract

To validate the feasibility of using next-generation sequencing in an African context, the complete genome of a rabies virus isolated from a human patient was obtained by high-throughput sequencing after virus isolation in mice and random unbiased amplification. Phylogenetic analysis suggested that this virus belongs to the Africa II clade.

## GENOME ANNOUNCEMENT

Although it is a fatal but preventable disease, rabies is still a widespread neglected zoonosis that represents an important public health threat in developing countries (1). Rabies virus (RABV) belongs to the genus *Lyssavirus* and family *Rhabdoviridae*. Humans get infected through close contact with secretions of rabid animals, mainly dogs. Rabies is almost always fatal once symptoms appear. Annually, 55,000 people die of rabies mostly in Asia and Africa (2). However, only a few complete genomes of African canine RABV are available (3, 4). Recent development of next-generation sequencing technologies allows virus whole-genome sequencing at relatively low cost. This might bring new insight into virus evolution, host-virus interactions, and pathogenicity (5). In September 2011, a man deeply bitten by a dog a few weeks before and suffering from mental confusion was treated at the anti-rabies dispensary in Bangui, Central African Republic, by 2 doses of vaccine according to the WHO-approved 2-1-1 regimen (6). Although required after severe exposures, rabies immunoglobulin was not administered as risk assessment was made difficult by incomplete anamnesis. He was supposed to come back for 2 more doses but never showed up again. Attempts to find out what happened remained unsuccessful. A sample of saliva was produced just before the first vaccine doses. Intra-cerebral inoculation of mice followed by direct immunofluorescence and PCR confirmed RABV presence in saliva. For the whole-genome sequencing, RNA extracted from inoculated mouse brain tissue using a QIAmp Viral RNA Minikit (Qiagen) was treated with TURBO DNAse (Life Technologies) to remove contaminating DNA and retro-transcribed using SuperScript III enzyme and random hexamers (Life Technologies). Amplification was done using Phi29 enzyme as described previously (7). High-throughput sequencing was performed by GATC Biotech (Konstanz, Germany) using a HiSeq 2000 system (Illumina). After removal of low-quality reads, 45.7 million of 100-bp single-reads were filtered using Bowtie 2 to discard murine sequences (8). Reads corresponding to RABV were selected by a similarity approach using BLASTn and BLASTx search tools with accession no. KC196743 as a reference (3). Final assembly was made using Ray software (9). Each position was covered on average 7,800 times. Overall length was 11,923 nucleotides (nt). Coding sequence lengths were 1,353 nt for the nucleoprotein, 894 nt for the phosphoprotein, 609 nt for the matrix protein, 1,575 nt for the glycoprotein, and 6,384 nt for the polymerase genes. Only one similar complete genome (98% identity) was found in GenBank after a similarity search, corresponding to the DRV-NG11 virus isolated from a dog in Nigeria in 2011 (3). Sequence analysis of the N gene showed a close relationship with viruses found in Chad and Niger in 1987, 1990, 1996, and 2005–2006 (identity ≥99%). Phylogenetic analysis suggests that our isolate belongs to Africa II clade group e, which includes viruses from Chad, Niger, Cameroon, and Nigeria (10). To our knowledge, this is the first reported complete genome of a RABV isolated from a human in Africa and one of a few of all the hosts combined.

### Nucleotide sequence accession number.

The complete genome sequence is available in the DDBJ/EMBL/GenBank database under accession no. KF977826.
